# A 5-Year Satisfaction Outcome Study of Patients Receiving Six-Implant-Supported Fixed Prosthesis

**DOI:** 10.3390/clinpract11040097

**Published:** 2021-11-02

**Authors:** Mazen A. Almasri

**Affiliations:** Oral Maxillofacial Surgery Department, Faculty of Dentistry, King Abdulaziz University, Jeddah 80209, Saudi Arabia; malmasri@kau.edu.sa

**Keywords:** edentulous, maxillary and mandibular arches, implant-supported fixed prosthesis

## Abstract

The aim of the study was to analyze the satisfaction of patients treated with a protocol of six-implant-supported fixed prosthesis (6IFP) throughout 5 years of service. This retrospective study collected the data of all patients who had full-arch rehabilitations using 6IFP and followed them for 5 years. After applying the research inclusion/exclusion strategy, 37 cases were finally included in the study. All the patients had no previous complete dentures because they were partially edentulous, not interested in pursuing complete denture rehabilitation, had immediate dental extractions, implantation used the 2-stage protocol, and there was minor peri-implant socket grafting. Cases with severe bone loss that required extensive grafting were excluded. A total number of 222 implants were placed in the maxillary or mandibular arches in a total of 37 patients. The data presented the satisfaction outcomes concerning mastication, phonetics, and comfort during the first 5 years of the recall plan. The former was achieved based on the clinical record reviews, follow-up visits, and recall phone calls at the preoperative stage as well as annually thereafter. The mean satisfaction rate was 94.5%, with a mean record of 8.21 ± 1.7 out of 10, there was no gender predilection significance, and no age range variation significance was validated. Regarding the smoking status, the t-test score exhibited no significant effect on phonetics and mastication (*p* = 0.12, *p* = 0.16, respectively), whereas comfort was found to be significantly affected (*p* = 0.03). The comfort level was found to be slightly less at the immediate postoperative period among smokers when compared to non-smokers. In conclusion, partially edentulous patients who received the rehabilitation plan of arch dental extractions, six immediate implantations, and delayed prosthetic loading were found to be highly satisfied.

## 1. Introduction

With the increase in personal healthcare awareness and digital promotion of self-care on the forums of social media and telecommunication, more care is being directed to general cosmetics including the smile, the face, and oral care. The field of oral rehabilitation provides variable options to patients with poor dental status and planning to pursue major refurbishing. Variable options are available for partially edentulous and completely edentulous patients, starting from restoring oral functions using removable dentures or utilizing dental implants for fixed prostheses [1,2]. Each treatment line has its pros and cons that need to be discussed with the patients to ensure the most suitable treatment that aids in building a solid professional relationship with the health care provider and to prevent any untoward misinterpretations or dissatisfaction [1]. Implant-based prosthetics may vary in implant number and prosthetic design. It has been advocated that more implants with fixed prosthetic designs would provide higher satisfactory results by fulfilling the parameters of the oral-health-related quality of life (OHRQoL) tool, such as mastication, phonetics, aesthetics, and general comfort [2,3].

A systematic review has evaluated the satisfaction rate of patients before and after the rehabilitation phase of using implant-supported fixed complete dentures (IFP) and overdentures [4]. The results showed that edentulous patients’ general satisfaction and OHRQoL is improved after receiving implant-based overdentures, and is even better after utilizing implant-fixed prosthesis [3]. The difference between the maxillary and mandibular arches has been reviewed, and the literature suggests that the mandibular arch is more challenging in which to achieve rehabilitation goals due to the arch mobility, tongue motion, limited keratinized gingival cover, and the decreased surface area when compared to the maxillary arch and palate [5]. It has been proven in a lot of studies that fixed prosthetics are preferable to patients when compared to conventional complete dentures and implant-retained prostheses, which coincides with our cases, because all of them refused to opt for a removable prosthesis despite the lower cost, and easier hygiene; therefore, they all elected to opt for implant-based rehabilitation. A study in Germany followed the patient satisfaction rate after using a screw-retained fixed prosthesis fabricated on six implants in the maxilla, over an 8-year period, which showed a cumulative implant survival rate of 99% and excellent prosthetic satisfaction among the patients [6].

The treating team must evaluate each case separately to customize the best management plan and minimize the gap between the patients’ preoperative expectations and the actual postoperative reality and satisfaction, especially regarding the fear of pain, treatment duration, cost, and the final prosthetic outcomes [7]. A discussion about the cost-effectiveness, the relationship to improvements in the oral health quality of life, and the further need for long-term recall visits must be included carefully while planning the treatment [8]. This study aimed to analyze the satisfaction level of partially edentulous patients with a poor dental status elected to choose a full management protocol that included complete dental extraction, immediate implantation with six implants, and further rehabilitation using 6IFP in the maxillary and mandibular arches throughout a five-year follow-up.

## 2. Materials and Methods

**Figure 1:**
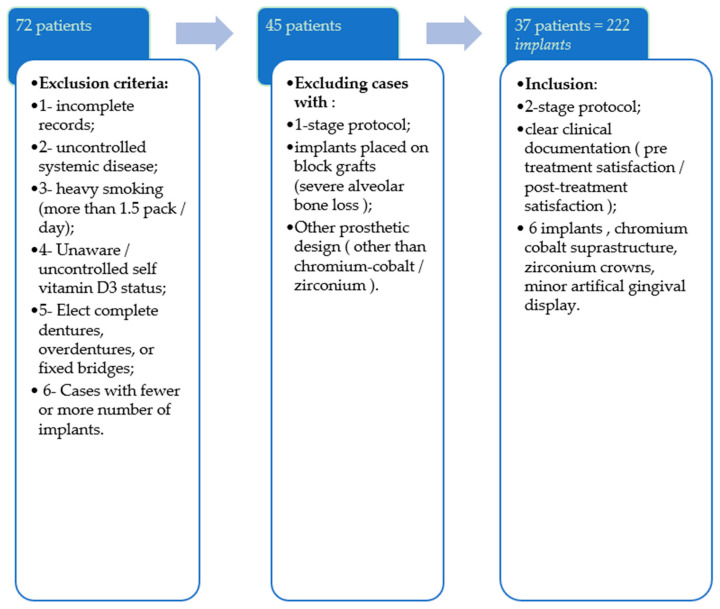
A flow chart presenting the case inclusion/exclusion strategy.

After receiving ethical approval from the institute’s research committee, a data review was performed that included all the cases that had had implant-based fixed prosthetics in the maxilla and mandible during the last seven years, from January 2010 until 2017. All the cases that were partially dentulous, indicated for complete extraction, refused complete dentures, and chose immediate implantation using the 2-stage implant-treatment protocol by placing 6 implants were included. The future rehabilitation option was a screw-retained fixed prosthesis, with a chromium cobalt supra-structure, zirconium crowns with an artificial gingival display (Figure 1). The pertinent information was collected for analysis; cases with incomplete surgical records, overdentures, or incomplete prosthetic details were excluded from the study.

The proper consultation interview took place with the oral maxillofacial surgeon and the prosthodontist, which included a thorough discussion of the stepwise plan, patient education by showing pictures, disclosing previous case outcomes, financial disclosure, offering multiple preoperative visits to answer any further questions, going through the consent form, and taking a preoperative record of the satisfaction level at the preoperative status. All the arches were planned for full dental extraction and immediate six-implant placement using the 2-stage protocol and minor socket grafting. The surgical procedures’ duration for each arch ranged from 30 to 60 min, prescribed careful postoperative antibiotics for five days, nonsteroidal anti-inflammatory (NSAID) analgesics, and oral hygiene instructions including Chlorhexidine 0.2% mouth wash to be used twice daily for one week. All the implants that were placed had a micro-threaded collar design (Astratech Ev^®^; Dentsply Sirona, and Nobel-Replace^®^; Nobel Biocare) with sizes ranging from 3.5 to 4.2 mm in diameter, placed at a moderately resorbed ridge that was managed successfully by immediate implantation and minor local socket grafting. No provisional prosthesis was loaded at any of the implants and temporary removable dentures were prescribed during the healing period. Further close follow-up visits took place to evaluate the recovery of the surgical sites. When the cases reached 4 months after implant placement, the second procedure stage took place, and the patients were referred to see the prosthodontist 2–3 weeks later to continue the care. The prosthetic design that was included in our study was composed of a screw-retained cobalt–chromium supra-structure, and zirconium crowns with an artificial gingival margin. Hence, cases of separate bridges or different denture materials were excluded from the study.

All the patient satisfaction feedback was perceived based on the recall interviews, phone calls, and the visual analog scale (VAS) recordings, where patients were asked about their phonetics, mastication ability, and the general comfort level, every year up to 5 years later. The primary period, which was from the time of prosthesis insertion up to 12 months post-insertion, was considered to be a trial and adjustment phase; hence, the satisfaction was assessed one year after the prosthetic placement and annually thereafter. The Oral Health Impact Profile (OHIP-14) questionnaire was partially used in our survey to help evaluate the perception. Four questions were utilized, and the grades ranged from 0 to 10. A preoperative baseline was recorded for all the patients. The results were graded as follows: “Strongly satisfied” was reported as “10”; “completely dissatisfied” as “0”; and “could not determine” was reported as “5”. Hence, the following questions were utilized:

1. How easily can you pronounce your words?
2. How much can you masticate your food (any difficulty/pain while eating)?
3. How comfortable and satisfied are you (less tension, less anxiety) with your prosthesis?
4. Do you have any further complaints?

The differences in the satisfaction level between the groups were analyzed by independent t-tests and Mann–Whitney U-tests using Statistical Product and Service Solution Ver.25 (SPSS-25). *p*-values of <0.05 were considered statistically significant.

## 3. Results

A total number of 222 implants were placed; two of them failed about 16 weeks after the placement, which was discovered during the second procedure stage without any apparent radiographical evidence or reason for treatment failure. Both belonged to patients with occasional smoking habits. The cumulative implant survival rate (CSR) was 99%. The failed implant sites were curetted and booked for reimplantation 6 weeks later, and the plan continued thereafter.

A total of 37 arches were treated with 6IFP; of which, 17 (46%) were mandibular and 20 (54%) were maxillary prostheses. There were 26 female patients and 11 males whose ages ranged from 31 to 88 years, and with a mean age of 58 ± 14.41 years. Most patients, i.e., 21 (56.8%), were in the age range of 61–70 years (Table 1).

Evaluation of the satisfaction level showed a mean score of 8.4 out of 10, which is considered an excellent grade. There were 14 (52%) patients who admitted to being moderate smokers, and 23 patients (48%) were non-smokers. The average satisfaction among smokers was found to be (8.21 ± 1.93) with regard to the mastication function, 8.21 ± 1.76 in phonetics, and 7.57 ± 2.68 with regard to the comfort. The comfort reported by the smoker group was found to be significantly lower than the total population and non-smoker records (Table 2).

The patients had an uneventful postoperative recovery time with no significant complaints, except for minor pain, which was tolerable after taking the prescribed non-steroidal anti-inflammatory drugs (NSAIDs) (25%). All cases attended their postsurgical recall visits, second procedure stage 4 months later, and started the prosthetic workup accordingly (Table 3). The satisfaction survey took place 12 months post-6IFP insertion, because the period before that was considered to be an adjustment period. The surveys continued annually thereafter.

When comparing male and female satisfaction using t-test, it was found that no significant differences existed (phonetics *p* = 0.39, mastication *p* = 0.6, comfort *p* = 0.68). Using the one-way ANOVA test showed that age was found to have no significance in correlation with the satisfaction of phonetics, mastication, or comfort (*p* < 0.05). Furthermore, the t-test showed that smoking was found to have no significant effect on phonetics (*p* = 0.12) or mastication (*p* = 0.160), whereas it was shown to have a significant correlation with comfort *p* = 0.03 (Table 4).

## 4. Discussion

The present study was performed in a standardized setting, had close recall visits, and an annual follow-up survey of 5 years to evaluate the satisfaction of the treatment process. Another study compared 86 patients who received IFP, removable implant prosthesis (RIP), and complete dentures [9], using OHIP-14. The results showed a significant difference in favor of the IFP and RIP when compared to the complete denture group (*p* < 0.05) [9]. One retrospective study included 40 patients who had a similar treatment protocol to the one used in our study of the 6IFP plan and were followed for 4 years. The study reported an implant survival rate of 98%, and 100% prosthetic success, except for minor issues that were managed conservatively such as loosening of the abutment screws, mucositis, or prosthesis minor fractures, which were encountered in a few cases in our series [10]. In our case protocol, peri-operative care was discussed thoroughly with the patient, including the delayed loading protocol and further recall visits needed. The implant CSR in our study was found to be 99%, which is slightly better than the study by Eliasson [11], where they found it to be 92–94.4% of implant survival rate in the early loading group compared to 98% at the delayed loading group; the result of a five-year recall study [10,11].

The satisfaction of phonetics with a functioning prosthesis has been compared and found to have superior performance with implant-based fixed prosthetics (a mean score of 8.3/10) when compared to “unsatisfactory” at the pre-operative phase. That was following another study reporting over 92% of pronunciation satisfaction post-implant-based rehabilitation [7,12]. Although the use of complete removable denture with maxillary full palatal coverage is considered to be a financially appealing design, when compared to implant-based prosthesis, it is correlated with a lower satisfaction record in phonetics, general comfort, and further gagging [13]. A thorough discussion of the stepwise treatment plan including the time frame is mandatory to be discussed including the possible complications. Even though it was reported that the satisfaction record started declining after any incidence of minor technical issues or prosthetic complications, such was not observed in our series and was managed conservatively [14,15].

Regarding mastication, the presence of implant-based prosthesis has been proven to be more satisfactory compared to conventional complete dentures in multiple studies. It has been reported that the use of implant-based prosthesis mainly improves the functions and quality of life, more than the nutrition intake itself [16,17].

There was no difference in gender predilection regarding satisfaction in this study. However, it was reported that males experienced a higher level of comfort, whereas females demonstrated more discomfort scores at the primary postoperative period and faded away thereafter. The survey showed that patients were comfortable after passing the primary prosthetic trial period, which accentuated the importance of discussing all the immediate and delayed case expectations including the comfort level, possible future gingival recession, and changes in the crown:gingival ratio [18]. On the other hand, smoking was a major barrier to the comfort satisfaction rate. The results in our study correlate with the literature which suggests that current or previous smokers have complications and discomfort during and after dental implant treatments [19]. Controversial studies claim the need for smokers to quit smoking 3–4 weeks pre-surgery, and the same post-surgery [19,20].

Although full-arch rehabilitation cases were reported among patients aged between 50 and 70 years [18,20], in our study, the age ranged from 30 to 88 years old and was found to have no significant effect on satisfaction. A single patient in our series, who was the eldest among the group and was known to be a smoker with moderately stable chronic obstructive pulmonary disease (COPD) had reported a generally poor satisfaction. This agrees with previous reports, suggesting that older patients might show lower satisfaction levels due to aging, a lower pain threshold, lower patience tolerance, and chronically compromised dental and medical conditions. This suggests the need for special care toward the elderly in general, and specifically while enduring a prolonged and possibly exhausting implant-based rehabilitation plan [20,21,22].

Investigating the factors controlling satisfaction is tedious work due to the multiple variables that might contribute, such as the level of education, age, medical health, type of surgical intervention, patience tolerance level, pain tolerance level, and the expertise of the treating team, including the postoperative maintenance plan [21,23,24]. In our series, a few of the aforementioned factors were considered, such as the expertise of the team, the constant recall plan, the surgical design, and the prosthetic design. Further studies will be needed to assess the included and excluded variables and their possible postoperative effects. As part of the recall plan, all the cases had an easily continuous communication line with the implant center through either phone calls or regular recall visits (every 3–6 months). This facilitated answering any queries, evaluating the hygiene, and addressing any peri-implant mucositis or discomfort. Furthermore, it has been stated that the relationship between the patient and the operator plays a significant factor in the satisfaction of the treatment and the future long-term follow-up, which was easily facilitated for our patients [18]. This study presents the experience of the implantology center in evaluating the satisfaction outcome of clients who had had 6IFP for full-arch rehabilitation and is presented to be a very satisfactory treatment protocol.

Concerning smoking, a significant area in the field of dental implantology, it was found that smokers of more than 20 cigarettes per day were found to have a relatively higher risk of failure, and hence will need to disclose such habits at the primary planning phase. It is recommended to refrain from smoking at least 2–3 days before and after the surgical date, to assure greater oral hygiene care, and attend all the recall visits to help control the peri-implant marginal bone and avoid mucositis as much as possible [25]. The two implants that failed belonged to two smoking patients with no clear reason indicating the loss of osseointegration in both of them.

Considering the tedious work to collect the details of the trial, the study does have some limitations, such as its retrospective nature. A prospective trial with a controlled group might help compare two or three different rehabilitation lines. Some variables could be included in future work to help evaluate the effects, such as heavy smoking, patients’ level of education, and different prosthetic materials.

## 5. Conclusions

This study presents a management protocol of full-arch rehabilitation using a six-implant-based fixed prosthesis, placed as late loading on immediate implants [26]. The results showed an excellent subjective satisfaction rate among the patients. The mastication, phonetics, and general comfort were found to be very satisfactory among the cases. The only difference was observed in smokers that exhibited satisfactory comfort levels; these were lower than in the non-smoking patients. Postoperative pain was found to be tolerable among all the cases. This indicated that the treatment protocol presented is a successful tolerable protocol, and we believe that the proper pre-, intra-, and postoperative detailed care supports the successful outcome [26].

## Figures and Tables

**Table 1:** Demographics of patients.

Variable		N (%)	Range	Mean ± Standard Deviation
Gender	Male	11 (29.7%)	-	-
	Female	26 (70.3%)		
Age	30–40	6 (16.2%)	31–38	58 ± 14.41
	41–50	5 (13.5%)		
	51–60	2 (5.4%)		
	61–70	21 (56.8%)		
	71–88	3 (8.1%)		

**Table 2:** Satisfaction among patients on a VAS out of 10.

Variables	N	Mastication	Phonetics	Comfort
		Response Mean ± Standard Deviation		
All patients	37	8.65 ± 1.48	8.68 ± 1.42	8.43 ± 1.94
Smokers	14	8.21 ± 1.93	8.21 ± 1.76	7.57 ± 2.68
Non-Smokers	23	8.91 ± 1.08	8.66 ± 1.11	8.96 ± 1.06
*p*-value	-	(0.160)	(0.12)	(0.03)

**Table 3:** Post-surgical complaints.

Response	Frequency (Percentage)
No complaint	33 (89.2%)
Tolerable pain	3 (8.1%)
Prolonged treatment time	1 (2.7%)

**Table 4:** Satisfaction among patients on responses out of 10 based on gender.

Variables		N	Response Mean ± Standard Deviation	*p*-Value
Smoking	F	26	2.08 ± 0.69	0.03
	M	11	1.45 ± 0.52	
Mastication	F	26	8.73 ± 0.96	0.06
	M	11	8.45 ± 2.34	
Phonetics	F	26	8.81 ± 0.98	0.39
	M	11	8.36 ± 2.16	
Comfort	F	26	8.54 ± 1.63	0.68
	M	11	8.18 ± 2.60	

## Data Availability

Available upon request.

## Abbreviations

OHRQoL	Oral-Health-Related Quality of Life
IFCDs	Implant-supported fixed complete dentures
IODs	Implant overdentures
VAS	Visual analog scale
SPSS	Statistical Product and Service Solution
CSR	Cumulative implant survival rate
NSAIDs	Non-steroidal anti-inflammatory drugs
COPD	Chronic obstructive pulmonary disease
